# The role of filamentation in activation and DNA sequence specificity of the sequence-specific endonuclease SgrAI

**DOI:** 10.1042/BST20220547

**Published:** 2022-12-16

**Authors:** Dmitry Lyumkis, Nancy C. Horton

**Affiliations:** 1 The Salk Institute for Biological Studies, La Jolla, CA 92037, U.S.A.; 2 Department of Integrative Structural and Computational Biology, The Scripps Research Institute, La Jolla, CA 92037, U.S.A.; 3 Graduate School of Biological Sciences, Section of Molecular Biology, University of California San Diego, La Jolla, CA 92093, U.S.A.; 4 Department of Molecular and Cellular Biology, University of Arizona, Tucson, AZ 85721, U.S.A.

## Abstract

Filament formation by metabolic, biosynthetic, and other enzymes has recently come into focus as a mechanism to fine-tune enzyme activity in the cell. Filamentation is key to the function of SgrAI, a sequence-specific DNA endonuclease that has served as a model system to provide some of the deepest insights into the biophysical characteristics of filamentation and its functional consequences. Structure-function analyses reveal that, in the filamentous state, SgrAI stabilizes an activated enzyme conformation that leads to accelerated DNA cleavage activity and expanded DNA sequence specificity. The latter is thought to be mediated by sequence-specific DNA structure, protein–DNA interactions, and a disorder-to-order transition in the protein, which collectively affect the relative stabilities of the inactive, non-filamentous conformation and the active, filamentous conformation of SgrAI bound to DNA. Full global kinetic modeling of the DNA cleavage pathway reveals a slow, rate-limiting, second-order association rate constant for filament assembly, and simulations of *in vivo* activity predict that filamentation is superior to non-filamenting mechanisms in ensuring rapid activation and sequestration of SgrAI’s DNA cleavage activity on phage DNA and away from the host chromosome. *In vivo* studies demonstrate the critical requirement for accelerated DNA cleavage by SgrAI in its biological role to safeguard the bacterial host. Collectively, these data have advanced our understanding of how filamentation can regulate enzyme structure and function, while the experimental strategies used for SgrAI can be applied to other enzymatic systems to identify novel functional roles for filamentation.

## Introduction

Filamentation of central metabolic and biosynthetic enzymes such as acetyl-CoA carboxylase, glutamate dehydrogenase, phosphofructokinase, and glutamine synthetase, among others, was first observed decades ago using electron microscopy to visualize enzymes purified from their native sources [1–18]. However, it was unclear whether enzyme filamentation was biologically relevant, and what, if any, effect it had on enzyme activity. The development of X-ray crystallography to study enzyme structure and function led to high-resolution snapshots of some of these enzymes in distinct structural states, including their interactions with substrates and products [19–24]. However, only some structural states, and not all enzymes, could be captured within crystalline forms. Filaments, due to their inherent structural heterogeneity, are less likely to crystallize. Hence enzyme filamentation was largely lost from the general consciousness of enzymologists and structural biologists. In the past decade, this subject has been revisited, in part due to dedicated efforts by individual laboratories studying enzymes that also happen to filament [25–30], advances in cryo-EM methods [31,32] and processing strategies for filamentous (typically helical) specimens [33,34], and the unexpected discovery of the formation of large, micron-scale, reversible self-assemblies (also referred to as filaments or cytoophidia in the literature) of many metabolic enzymes in cells [35–42]. These self-assemblies tend to form in cells only under particular conditions, often during nutrient depletion or other types of stress, and their discovery sparked renewed interest in self-assembly of enzymes and its possible involvement in enzyme regulation.

SgrAI is a homodimeric sequence-specific DNA endonuclease from the type II restriction endonuclease class that exhibits unusual DNA cleavage activities [43]. SgrAI cleaves two types of recognition sequences in DNA, known as primary and secondary sequences. The enzyme cleaves primary sequences slowly when they are present as single copies on a DNA molecule such as a plasmid, but up to 200 times faster when they are present in more than one copy [43]. The enzyme cleaves secondary sequences only when they are present on the same molecule of DNA as a primary sequence [43]. Hence the presence of the primary sequence activates SgrAI and expands its DNA cleavage activity to include additional secondary sequences. There are three primary sequences, following the pattern CR|CCGGYG (| denotes cleavage site, Y = C or T, R = A or G), and fourteen secondary sequences following the patterns DRCCGGYG (where D = A, G, or T) or CCCCGGYG, giving a total of seventeen sequences cleaved by SgrAI in the activated state [43]. Through a series of biochemical, biophysical, and structural analyses, the activated state of SgrAI was demonstrated to be filamentous [25,44,45], formed by the assembly of DNA bound SgrAI dimers (or DBDs, ∼100 kDa each) into a left-handed helix, which is held together largely by interactions between SgrAI enzymes, with the bound DNA looping in and out of the filament [45]. Only primary sequences will stimulate filament formation by SgrAI, whereas secondary sequences will not. However, SgrAI bound to secondary sequences will join into filaments formed by SgrAI bound to primary sequences. This filament could, in principle, extend infinitely, but in both experimental and biological settings is expected to be limited to sizes of twenty DBDs or less [45]. Detailed studies on the structure, kinetic mechanism, and biological role of SgrAI filamentation have shed light on how filamentation regulates this enzyme’s behavior.

## Discovery of filament formation by SgrAI

The unusual DNA cleavage activities of SgrAI suggested that its primary recognition sequence can serve as both a substrate, which is cleaved by SgrAI, as well as an allosteric effector, which activates SgrAI and also modulates its sequence specificity to extend cleavage to secondary sequences [25,43,46]. However, early biophysical and structural studies demonstrated only a single DNA binding site on the DBD [25,47,48]. Because SgrAI binds both primary and secondary DNA sequences tightly, the lack of cleavage of secondary sequences, in the absence of primary sequences, could not be explained by the lack of SgrAI binding to secondary sequences [25]. Therefore, a plausible model that explains how SgrAI cleaves one type of sequence (i.e. primary or secondary) by the binding of another (i.e. a primary sequence) would involve at least two copies of SgrAI DBDs forming a higher-order assembly; in this model, the SgrAI DBD bound to a primary sequence activates the DNA cleavage properties of the SgrAI DBD bound to either a primary or secondary sequence [47,49].

The smallest higher-order assembly that fits the cooperative cleavage model is a dimer of SgrAI dimers, i.e. 2x DBDs. However, numerous biophysical studies, including native gel electrophoresis, analytical ultracentrifugation, and ion mobility native mass (IM-MS) spectrometry suggested that the activated enzyme form was composed of an average of 12 DBDs, with sizes ranging from 2–30 DBDs [25,44]. Mutagenesis of residues residing far from the active sites, which do not immediately mediate DNA binding and cleavage but do disrupt the formation of larger assemblies, clearly disrupted enzyme activation as well [25]. These experiments provided supporting evidence that the activated species is the heterogeneous large assembly. IM-MS analyses indicated that activated SgrAI species differed in size by increments of single DBDs. Hence, the DBD was identified as the basic building block of the activated oligomeric SgrAI species [44]. Furthermore, the collisional cross sections determined by IM-MS were found to increase linearly with an increasing number of DBDs, suggesting that the activated species was composed of a regular, repeating structure [44]. One way to assemble a collection of species that is characterized by specific structural features (which would explain the special enzyme activity) but that is heterogeneous in mass is through filamentation. Enzymes within a filament can exhibit the same structural conformation, but there are no restrictions on filament length. Filamentation would naturally lead to a collection of species of differing lengths, and therefore different masses.

**Figure 1.**
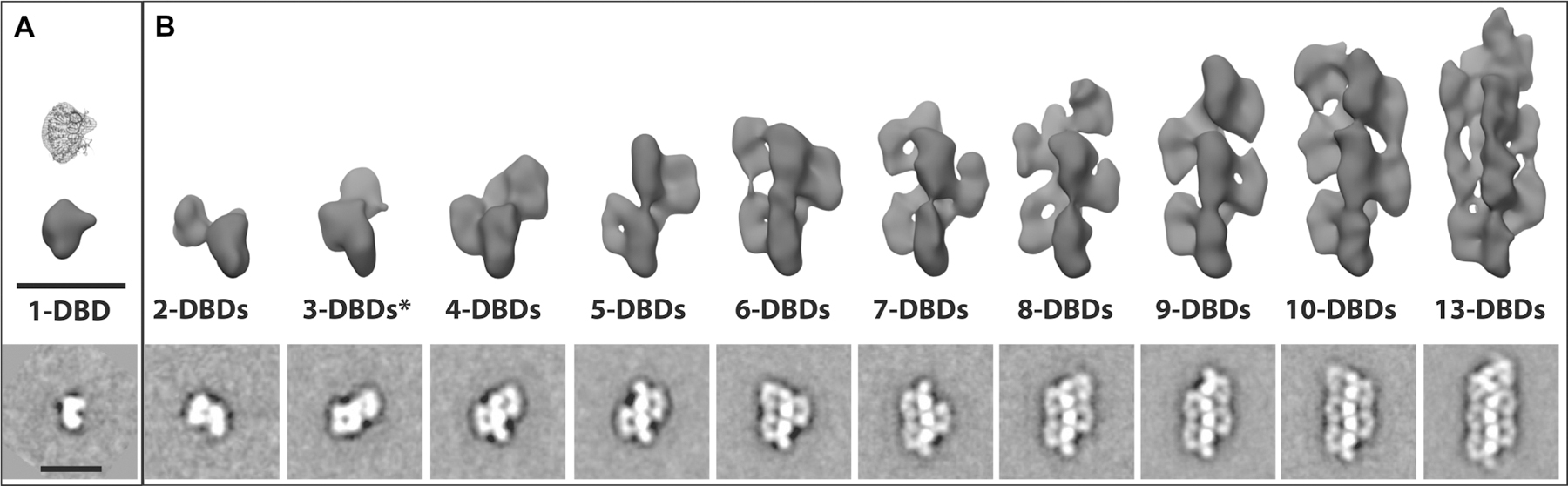
Characterization of run-on SgrAI filament formation by negative stain electron microscopy. (**A** and **B**) 2D class averages from negatively stained images of SgrAI (bottom) and corresponding reconstructed density maps generated using the random-conical tilt reconstruction strategy (top) for dimeric and filamentous SgrAI. (**A**) A single SgrAI DBD with the corresponding crystal structure [48] displayed above. (**B**) Oligomeric SgrAI assembled into filaments of increasing sizes ranging from 2-DBDs to 13-DBDs. From these reconstructions, it was possible to visualize the helical nature of the filament and approximate the helical parameters that were subsequently used for high-resolution refinement from cryo-EM data. Scale bars represent 150 Å. Figure from ref. [45].

**Figure 2.**
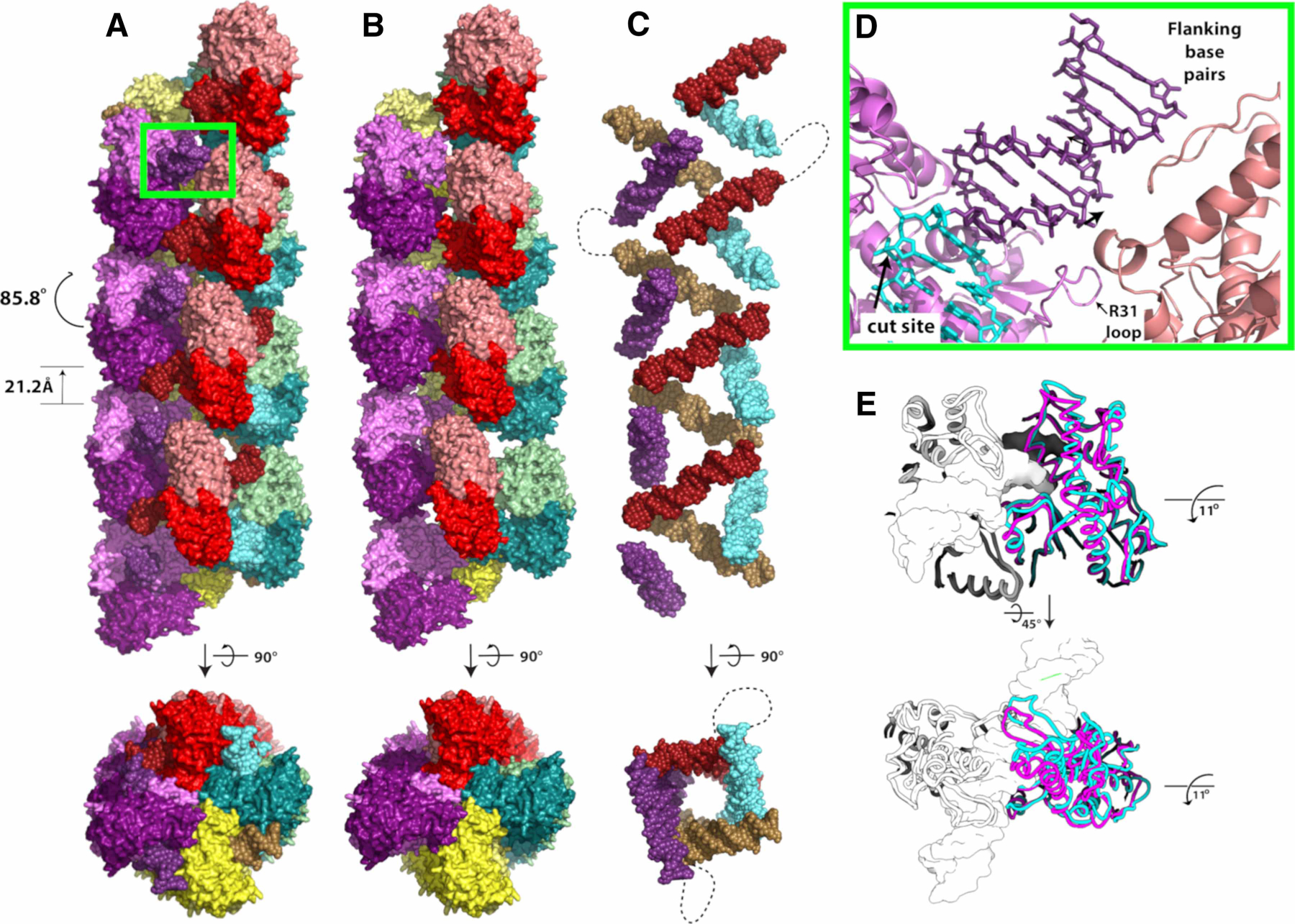
Filament of SgrAI bound to its primary recognition sequence and induced conformational change. (**A**) Left-handed helical filament formed from DNA bound SgrAI dimers (DBD, colored in red, green, purple or yellow). Approximately four DBDs are found per turn, with helical parameters −85.8° twist and 21.2 Å rise between subunits. Within each DBD, the DNA and two SgrAI chains are colored in slightly different hues. (**B**) As in **A**, but without the DNA shown. (**C**) As in **A**, but with only the DNA shown. The dotted line shows one way two duplexes could be connected within the filament. Note that within biologically relevant DNA, this loop may be ∼500–1000 bp in length. (**D**) Close-up of the region between adjacent DBDs of the green boxed region in A showing the position of the R31 loop between the red and purple DBDs, the position of the cleavage site, the primary recognition sequence (cyan), and bp flanking the recognition sequence that contact neighboring DBDs (salmon). (**E**) Comparison of DBD from filamentous (cyan) and non-filamentous (magenta) structural states reveals an 11° rotation of one chain of the SgrAI dimer relative to the other approximately parallel to the path of the DNA.

Confirmation of filamentation was obtained by electron microscopy of negatively stained DNA bound SgrAI (Figure 1) [45]. The sample contained SgrAI protein bound to a 40 bp oligonucleotide with a single primary sequence [25,43], and the resulting images showed a distribution of filament lengths composed of individual DBDs, as predicted by the earlier biophysical studies. The definitive identification of helical symmetry was obtained after performing 3D reconstructions of groups of negatively stained image averages using the random conical tilt reconstruction strategy [50,51] (Figure 1). In all cases, 3D reconstructions of classes corresponding to filaments containing increasing numbers of DBDs revealed a left-handed helical organization containing approximately four DBDs per helical pitch. Subsequent cryo-electron microscopy experiments led to a sub-nanometer resolution reconstruction of the ensemble averaged filamentous assembly. The structure confirmed the left-handed helical nature of the filament and revealed new contacts between adjacent DBDs within the filament (Figure 2A,B) [45]. The filament is not assembled linearly on one long DNA molecule. Rather, the filament is held together by both protein–protein interactions (Figure 2B) and protein–DNA interactions (Figure 2D) between adjacent DBDs. The DNA duplexes bound by SgrAI in the filament are displaced from the helical center and tilted ∼45° from the filament axis (Figure 2C). In a biologically relevant DNA molecule, the distance between SgrAI recognition sites is estimated based on the statistical occurrence within random DNA sequences (and as found in *Streptomyces* phage R4 and PhiC31) to be ∼500–1000 bp[52]. Hence, the DNA would enter and exit the filament, with loops ranging in length from ∼500–1000 bp. This is shown as dotted lines in Figure 2C. More recent structures extended the resolution of the first reconstruction, leading to refined helical parameters of 21.2 Å rise and −85.8° twist and confirmed the importance of enzyme-DNA contacts outside of the active site [53,54]. These contacts explain several earlier results, such as the importance of the length of DNA used to induce activation, since the ends of the 40 bp DNA make stabilizing contacts to neighboring DBDs, and how mutations disrupt filamentation at the amino-terminus of SgrAI, since neighboring DBDs interact in the filament via their amino-terminal residues [25,45].

## Structural mechanism of activation and expansion of specificity of SgrAI through enzyme filamentation

Structures of SgrAI have now been solved to high-resolution in multiple states, including without bound DNA [54], bound to primary or secondary sequences in the non-filamentous state [48,55], and bound to a primary sequence in the filamentous state [53,54]. Analysis of these available structures has led to a model for filamentation-induced enzyme activation and to several hypotheses for the special secondary sequence cleavage mechanism exhibited by SgrAI. The mechanism is thought to involve two Mg^2+^ ions bound at the protein–DNA interface near the site of cleavage; however, in the non-filamentous form, one of the two Mg^2+^ ions is distant from its optimal position [48,55], as expected for many divalent cation-dependent DNA endonucleases [56–58]. Since SgrAI cleaves DNA slowly in the absence of filamentation, the misplacement of this Mg^2+^ ion is proposed to be the origin of its low DNA cleavage activity in this conformation. Otherwise, the structures of SgrAI protomers in the non-filamentous form are nearly identical at the limit of resolution, regardless of whether primary or secondary site sequence is bound [48,55], indicating that defined structural changes are necessary to explain the enzyme’s activation.

**Figure 3.**
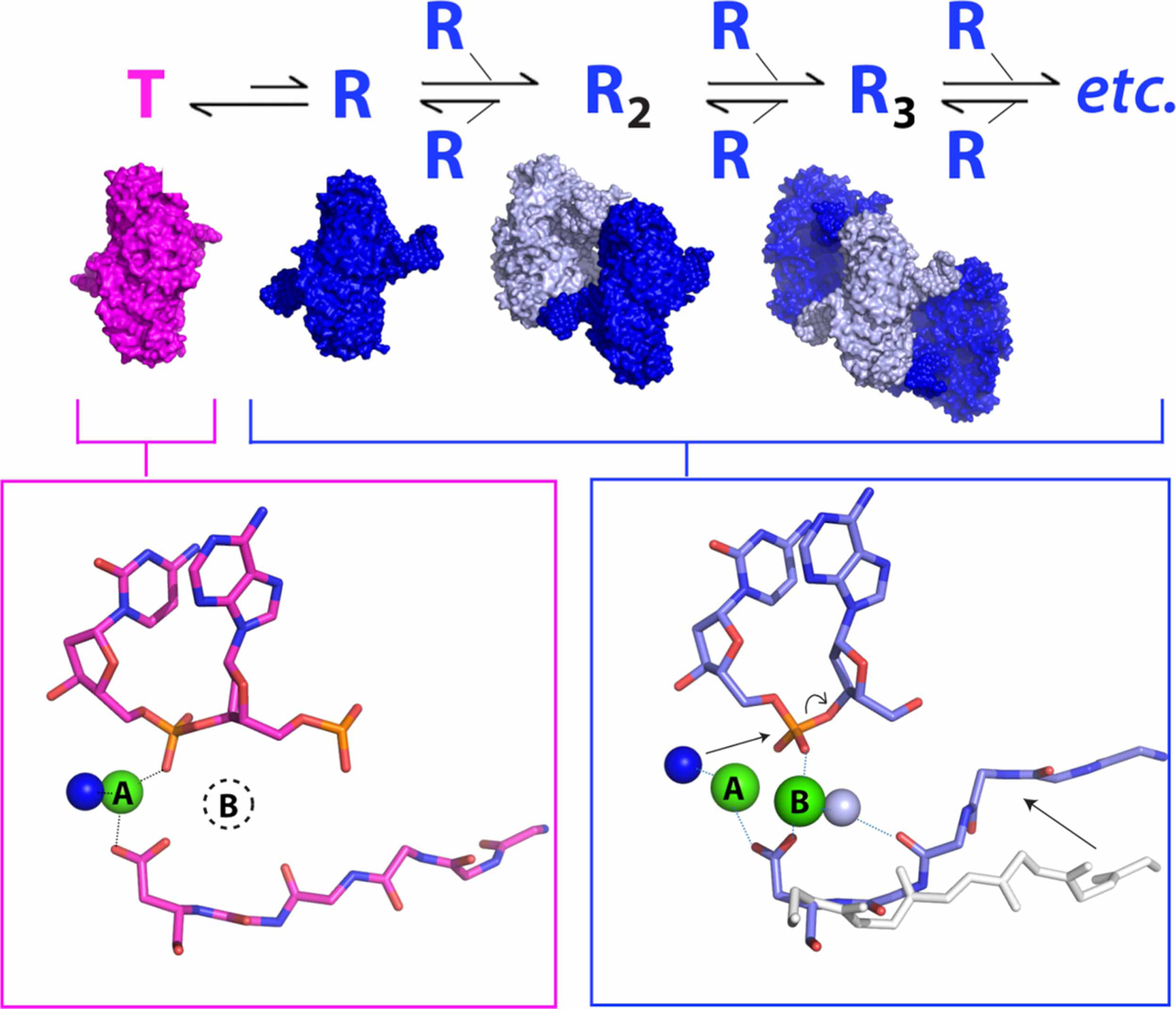
Allosteric equilibrium model of SgrAI filamentation and activation. SgrAI bound to its primary recognition sequence (DBD, pink, blue) is in equilibrium between a low-activity conformation (the T state, pink) and a high-activity conformation (the R state, blue). The T state structure is missing the second Mg^2+^ ion in the active site (dotted circle encircling the letter B in the pink box) and does not form filaments. The R state DBDs assemble into filaments with activated DNA cleavage activity, originating from occupancy of both Mg^2+^ ions in the active site (sites A and B). The conformational change stabilized by contacts between DBDs in the filament results in a shift of residues of SgrAI, which creates a high affinity binding pocket for the site B Mg^2+^ (right-most arrow within blue box) involving a bridging water molecule (light blue sphere). Mg^2+^ in site A ligates the nucleophilic water (dark blue sphere), which is responsible for attack on the phosphorus atom at the site of cleavage (left-most arrow within blue box), leading to bond breakage between the phosphorus atom and the O3′ (curved arrow within blue box). All atoms were modeled into and refined against experimental cryo-EM maps, including the water molecules.

In the filamentous state, contacts between neighboring DBDs stabilize a distinct conformation of SgrAI and its bound DNA that is specific to its activated form [54]. The global change in conformation involves the rotation of one protomer relative to the other by ∼11°, approximately parallel to the path of the DNA (Figure 2C). To accommodate this change, protein segments at the dimeric interface must shift, and this shift connects directly to the protein–DNA interface and the active site where DNA cleavage occurs. The shift stabilizes the binding of the second Mg^2+^ in the appropriate location for catalysis (Figure 3). The high-resolution of the experimental maps allowed the identification of important water molecules such as the putative nucleophile (dark blue, Figure 3) and a water molecule stabilizing the second Mg^2+^ ion (light blue sphere, Figure 3) within the active site. Hence, filamentation leads directly to changes in the active site and to the correct positioning of Mg^2+^ to facilitate rapid DNA cleavage [54].

To explain the expansion of DNA sequence cleavage specificity of SgrAI, which occurs upon filamentation, an allosteric model was developed involving the equilibrium between two distinct DBD conformations, a low activity T state as observed in non-filamentous structures, and a high activity R state as observed in filamentous structures [54] (Figure 3). In the absence of filamentation, the T state is favored, but the R state can be populated when particular conditions are met; at sufficient concentrations of enzyme and DNA, R state DBDs can assemble into filaments, which in turn stabilize the R state long enough to allow DNA cleavage to occur. When bound to primary sequences, the equilibrium between T and R states is shifted toward the R state to allow filamentation and cleavage to occur. In contrast, when bound to secondary sequences, the equilibrium is shifted more towards the T state. This explains the limited propensity of DBDs to form filaments when SgrAI is bound to secondary sequences, since the R state is rarely populated. However, the R state remains energetically accessible, even when SgrAI is bound to secondary sequences, which enables filamentation nucleated by SgrAI bound to primary sites and leads to a cleavage cascade. Hence, according to this model, cleavage of secondary sequences will occur appreciably only in the presence of SgrAI bound to primary sequences, which is observed experimentally [25,43,46,59].

The allosteric model implies differing stabilization of the T and the R states by the bp differences (and their interactions with SgrAI) in primary and secondary recognition sequences, with the T state being more stabilized with secondary sequences bound. To search for possible origins of this stabilization, structures representative of the T and R states were closely compared. To date, no structures of SgrAI bound to secondary sequences in the R state have been determined. There is one reported structure of SgrAI bound to a secondary sequence with the pattern CCCCGGYG in the non-filamentous T state, but it shows no conformational or structural differences with the T state structure of SgrAI bound to a primary recognition sequence at the limit of resolution (1.89 Å and 2.65 Å resolutions for SgrAI bound to primary and secondary site, respectively) [55]. Therefore, structures of T and R states of SgrAI bound to a primary recognition sequence were used to assess changes in structure that may be impacted by the substitution of bp in the first or second position of the recognition sequence, which are the variable positions. Two relevant observations were made: (i) a change in base stacking in the T and R states at the second bp, and (ii) a close approach of a segment of SgrAI to the first bp of the recognition sequence in the R state [54]. Base pair stacking stabilizes DNA structure and is sequence dependent [60–62]. A change in base stacking in the T and R states would impact the overall stability of each conformational state and depend on the base identities. An analysis of base stacking areas in the two states, and comparison to experimentally estimated stacking energies indicated that the substitution at the second bp of the recognition sequence, as found in CCCCGGYG, would shift the equilibrium between T and R states towards the T state [53].

Most secondary sequences cleaved by SgrAI exhibit the pattern DRCCGGYG. The close approach of an SgrAI segment to the outer ‘D’ bp substitution in secondary sequences could destabilize the R state when SgrAI is bound to such sequences [54]. In addition, filamentation of SgrAI when bound to these types of secondary sequences may further be disfavored due to a disorder-to-order transition in the segment of protein that contains the amino acid side chain that forms a sequence-specific hydrogen bond to the outer bp of the primary recognition sequence. This mechanism was proposed due to the observed disorder of this segment, known as the R31 loop, in the structure of SgrAI without bound DNA [54]. Since the segment is ordered when SgrAI is bound to primary sequences, it must undergo a disorder-to-order transition upon DNA binding, likely due, in part, to the interaction of the R31 side chain to the G base of the outer bp of the recognition sequence (i.e. the base pairing partner to the first C of the primary recognition sequence CRCCGGYG). When this bp is substituted and the G is replaced with A, C or T, the Arg side chain would not be able to form the same interaction, and the R31 loop may remain partially or completely disordered as a result. The ordering of this segment is critical to filamentation, since it forms an interface with neighboring DBD in the filament, contributing to filament stabilization (Figure 2B) [54]. Hence, SgrAI bound to secondary sequences containing the pattern DRCCGGYG should disfavor the R state conformation due to steric conflicts and disfavor the filament-competent R state with the ordered R31 loop due to the added entropic cost of the disorder-to-order transition.

## Kinetic mechanism of DNA cleavage by filamentous SgrAI

The proposed mechanism for accelerated DNA cleavage by SgrAI mediated through filamentation must account for the fact that SgrAI enzymes do not become trapped within filaments, which would result in only one cleaved DNA recognition sequence per enzyme dimer. In addition, since filament formation can be rate-limiting under certain contexts, the mechanism must explain the accelerated cleavage reaction observed under experimental conditions. The experimentally observed distribution of filament sizes favoring smaller lengths suggested that the filaments are in rapid equilibrium with the non-filamentous state. If true, how would DNA cleavage occur in the short timeframe when a DBD was within a filament? To answer these questions, global kinetic modeling was performed using data from single turnover DNA cleavage reactions combined with mechanistic modeling of each step in the reaction pathway [63–65]. Different models were built and tested for their fit to the experimental DNA cleavage data. Since each reaction step — filament formation, filament dissociation, and DNA cleavage — must be explicitly defined, the model becomes increasingly complex when accounting for all combinations and permutations of filaments of differing lengths. Thus, modeling was limited to filaments no longer than five DBDs. However, evidence suggested that filament sizes under the experimental conditions used largely remained within the accessible modeling range, and the data fitting resulted in well-defined rate constants for most steps of the reaction pathway [63,64].

Most importantly, rate constants for filament association from DBDs, DNA cleavage when in the filament, and filament dissociation into DBDs, were well determined by the global data fitting. The rate constant for DNA cleavage was found to be at least 10 times faster than the rate constant for dissociation of DBDs from filaments. However, the fitted dissociation rate constant is sufficiently fast to prevent ‘trapping’ of DBDs within the filament [64]. Hence the global fitting showed that DBDs assemble into filaments, cleave bound DNA, and dissociate to release the cleaved DNA and begin another round of DNA cleavage on biologically relevant time-scales. Another interesting observation was that the second-order rate constant governing filament formation was relatively slow, at least 1000 times slower than possible in the fastest of enzyme reactions (i.e. when controlled merely by the rate of diffusion and collision between reactants) [64]. At first glance this seems counter-intuitive; why evolve a slow, rate-limiting step in a mechanism used to accelerate an enzymatic reaction? However, simulations using the kinetic model with concentrations of recognition sites expected within the cell show that this slow step may be important for the biological function of SgrAI, as explained below [52].

## Biological advantages of enzyme regulation via filamentation

**Figure 4.**
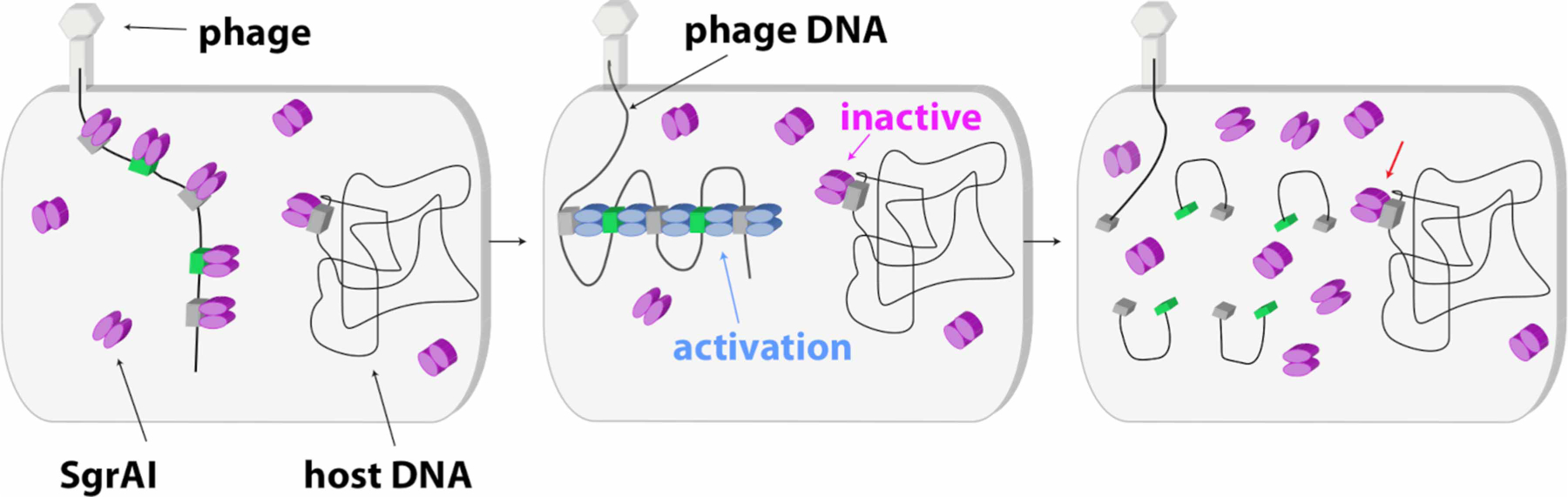
Model of SgrAI filamentation activated DNA cleavage. ***Left***: SgrAI dimers (magenta) bind to primary (green boxes) and secondary (grey boxes) sequences on both the invading phage DNA and the host DNA. Primary sequences on the host DNA are methylated and are not bound by SgrAI. SgrAI largely maintains the T state prior to filamentation ( pink ellipses). ***Center****:* SgrAI bound to the primary recognition sequence occupies the R state (blue ellipses) in sufficient concentrations to nucleate filaments drawing in and activating SgrAI bound to secondary sites on the phage DNA. SgrAI bound to secondary sequences on the host genome do not induce filamentation and are not drawn into filaments nucleated by SgrAI bound to the phage DNA because of the slow, second-order rate constant for filament assembly and the low concentrations of SgrAI bound DNA in the cell when on separate DNA molecules. ***Right****:* SgrAI cleaves primary and secondary sequences in the phage DNA only, leaving the host DNA unharmed (red arrow).

Simulation of SgrAI-mediated DNA cleavage in the cell using estimated biologically relevant concentrations of genomic recognition sites reveal that the slow second-order rate constant for DBD association into filaments controls where and when SgrAI becomes activated [52]. This is critical since secondary sequences are numerous and present throughout the host genome (*Streptomyces griseus*) but are not protected from cleavage. Primary recognition sequences are protected from cleavage due to the action of the SgrAI methyltransferase, which methylates the C at position 3 of the recognition sequence (CRCCGGYG), preventing enzymatic cleavage. Hence, the many secondary sequences on the host genome are susceptible to damaging DNA cleavage by activated SgrAI. Activation of SgrAI is prevented by the absence of competent (i.e. non-methylated) primary sites on the host genome, since only binding to non-methylated primary sites can induce filamentation and thereby enzyme activation. However, invading DNA, such as from bacteriophage, contain unmethylated primary recognition sequences that will activate the enzyme (Figure 4). The high local concentrations experienced by recognition sites on the same molecule of DNA overcomes the slow second-order rate constant for filamentation, and SgrAI bound to both primary and secondary sequences on the same DNA molecule will therefore assemble into activated filaments. SgrAI bound to secondary sites on the host genome do not become activated, since the slow, second-order rate constant for filament assembly, combined with the actual concentrations of the DNA sequence, now on separate molecules, ensures slow and/or negligible assembly (Figure 4) [52]. The filament lifetime is too short to result in significant DNA damage to the host. However, such a sequestering effect could in principle also be accomplished by assembly into any sort of oligomer, not just filaments. Why would DBDs assemble into filaments rather than closed oligomers such as a dimer of DBDs (i.e. a binary system)? Side-by-side comparison using simulations of DNA cleavage by the filament versus a binary mechanism shows that filamentation leads to a faster overall rate of DNA cleavage [66,67]. The origin of this superior acceleration lies in the multiple ways filaments may assemble, including at either end, making collisions between molecules more likely to result in productive assembly of DBDs into activated forms.

In vivo tests of the filamentation mechanism were performed using a phage protection assay and mutations in SgrAI that disrupt, to different extents, SgrAI filamentation, but without disrupting other aspects of the DNA cleavage mechanism [52]. WT and mutant SgrAI enzymes were expressed in a bacterial system and challenged with phage. The result was surprising in that only WT SgrAI, and one mutant form with no measurable disruption to filamentation, could protect bacteria from phage infection. All other mutant SgrAI, even those that could still, to a lesser degree, assemble into filaments (three-fold reduced) gave little to no protection from phage infection [52]. Hence, the rapid kinetics of DNA cleavage by SgrAI appears critical to the enzyme’s biological function. SgrAI must outpace phage replication and also the action of the host methyltransferase, since methylation of primary sequences on the phage DNA would render it immune to cleavage.

## Filament-forming enzyme mechanisms

Our studies have shown that filamentation activates the DNA cleavage activity of SgrAI. Because of the differences with respect to nucleation of filamentation between the two types of recognition sequences, filamentation effectively expands the DNA sequence specificity of SgrAI from only primary sequences to additionally include secondary sequences; the number of recognition sequences increases from from 3 to 17 in total. Other filament-forming enzymes include the well-known actin and tubulin proteins. Similarities with SgrAI include the activation of enzyme activity (ATPase or GTPase, respectively) through filamentation [68,69], but there are also important differences related to the polarity of the filaments and their stability following the ATP or GTP hydrolysis. Both actin and tubulin are polar, growing faster from one end than the other [70,71], whereas the ends of SgrAI are identical, meaning that filament growth is expected to be equivalent at either end. Actin and tubulin filaments also weaken following nucleotide hydrolysis [72,73], whereas there is currently no evidence that DNA cleavage weakens the affinity of SgrAI DBD to the filament. Although enzymatic activity is accelerated in all three enzymes as a result of filamentation, that in the cytoskeletal filaments is much slower and is used as a ‘timekeeper’ to control filament length and lifetime [74], whereas the DNA cleavage rate of SgrAI is accelerated specifically to rapidly cleave invading phage DNA.

Many other enzymes form filaments, with varying consequences to their activity. The enzyme acetyl CoA-carboxylase (ACC), which plays an important role in fatty acid synthesis, is now known to form three different types of filaments, one stabilizing an active conformation and two stabilizing inactive conformations [75]. The high-resolution structures of filaments of the nucleotide synthesis enzyme cytosine triphosphate synthase (CTPS) from four different organisms (yeast, human, fruit flies, and bacteria) have now been characterized [76–80]. The consequence to enzyme activity of CTPS varies: filamentation inactivates CTPS activity in *E. coli* and yeast [76,80], but activates the human CTPS1 isoform [77]. A second human isoform, CTPS2, forms filaments of either the active or inactive forms (but not both simultaneously), and rapid and cooperative interconversion between the two states is made possible by their close proximity in the filament [81]. These examples demonstrate how filamentation can either lock an enzyme into one type of conformation, thereby controlling activity simultaneously using many enzyme copies, or alternatively quickly and cooperatively shift from one conformation to another, rapidly inducing activation or inactivation as needed [81]. Another important enzyme in nucleotide biosynthesis is inosine monophosphate dehydrogenase (IMPDH), which forms filaments capable of accommodating both the active and inactive conformations. However, filamentation appears to decrease the responsiveness of the enzyme to inhibition by the feedback inhibitor GTP, and hence may function to buffer against transient changes in metabolite concentrations [82,83]. The enzyme AdhE, which catalyzes the conversion of acetyl-CoA to ethanol during bacterial fermentation, forms filaments with channels between different active sites in its two-step pathway, increasing the rate of transfer of intermediates and preventing the release to the cytoplasm of potentially toxic molecules [84,85]. Finally, closely related plant nitrilase enzymes form filaments with slightly different helical twists, resulting in different binding site shapes and therefore altered substrate specificities [86]. These are only a few examples, and the effects on enzymatic activity of many additional enzymes have yet to be discovered and are an important and active area of research [17].

## Summary

The host organism of SgrAI, *Streptomyces griseus* (*S. griseus*), possesses a significantly larger genome than other bacteria, which may explain the unusual behavior of its innate immune system defined by the SgrAI enzyme. Restriction-modification (RM) systems, in general, protect their host from invading DNA by cleaving specific sequences within the invading DNA, thereby incapacitating their replication. Specific DNA sequences must be recognized, because random DNA cleavage would make protection of the host DNA difficult (which is not separated from the cytoplasm by a nucleus in bacteria). The cognate methyltransferase of the RM system (i. e. SgrAI.M) protects the host DNA by methylating the shared recognition sequence (i.e. the primary sequence), thereby protecting it from cleavage by the cognate endonuclease, here SgrAI. With the large genome of *S. griseus*, many more copies of the recognition sequence will be present, which must be methylated to be protected from cleavage. This protection comes at a cost, since methylation requires the cofactor S-adenosylmethionine (SAM). Perhaps this is why the SgrAI endonuclease possesses a low intrinsic DNA cleavage rate and a relatively longer, and therefore rarer, recognition sequence (8 bp instead of the more typical 4–6 bp). However, both these characteristics, which protect the host from SgrAI, also decrease SgrAI-mediated anti-viral activity, since fewer recognition sites and a lower DNA cleavage activity could allow invading phage to escape restriction and replicate out of control, ultimately killing the cell. But SgrAI becomes activated by the presence of the invading DNA through binding to its primary recognition sequences and forming a filament stabilizing its activated conformation. It also expands DNA sequence specificity by allowing SgrAI bound to secondary sequences to be drawn into the filament, thereby activating them for DNA cleavage. Secondary sequences on host DNA are protected by this less discriminating, activated SgrAI since the slow second-order rate constant for filament assembly prevents their inclusion into filaments (Figure 4).

## Perspectives

- More than 30 enzymes from distinct enzymatic pathways and cell types (bacteria, yeast, and metazoans) are known to form filaments exhibiting a diversity of architectures, including both helical and linear filaments, single, double, or triple helices, and in some cases, cylindrical tubes. Many more enzymes can form large self-assemblies in cells with unknown architectures, possibly as a result of enzyme filamentation, and understanding the principles governing filamentation provides opportunities to discover novel mechanisms by which enzymes are regulated and strategies to modulate enzyme activity.
- The SgrAI enzyme is one of the best-studied model systems among filament-forming enzymes, and biochemical, biophysical, and structural biology studies have provided key insights into the functional implications of filamentation in SgrAI’s role to defend its host against invading phage. The functional role of filamentation by many other enzymes remains unknown, but studies to date have suggested that filamentation can modulate enzyme activity, regulate enzyme cooperativity, specificity, or sensitivity to allosteric effectors, affect signaling cascades, control cellular differentiation and localization, protect from stress, tune cellular metabolism, among other functions.
- The field involved with studies of filament-forming enzymes, exemplified here with the SgrAI system, is rapidly developing, and it is likely that many more filament-forming enzymes, and the roles that filamentation play in regulating enzyme activity, will be revealed. The relationship between large cellular self-assemblies (also known as cytoophidia, rods and rings, and macrofilaments) and enzyme filaments, in most cases, has yet to be defined, but over 100 enzymes are known to form such structures in cells. Modulation of filamentation by medically relevant enzymes is likely to provide new avenues for therapeutic intervention.

## Abbreviations

CTPS: cytosine triphosphate synthase
IM-MS: ion mobility native mass
RM: restriction-modification
